# Protective Effects of the Mushroom *Lactarius deterrimus* Extract on Systemic Oxidative Stress and Pancreatic Islets in Streptozotocin-Induced Diabetic Rats

**DOI:** 10.1155/2015/576726

**Published:** 2015-06-29

**Authors:** Mirjana Mihailović, Jelena Arambašić Јovanović, Aleksandra Uskoković, Nevena Grdović, Svetlana Dinić, Senka Vidović, Goran Poznanović, Ibrahim Mujić, Melita Vidaković

**Affiliations:** ^1^Department of Molecular Biology, Institute for Biological Research, University of Belgrade, Bulevar Despota Stefana 142, 11060 Belgrade, Serbia; ^2^Department of Biotechnology and Pharmaceutical Engineering, Faculty of Technology, University of Novi Sad, Bulevar Cara Lazara 1, 21000 Novi Sad, Serbia; ^3^Biotechnical Faculty, University of Bihać, Kulina Bana 2, 77000 Bihać, Bosnia and Herzegovina

## Abstract

The aim of this study was to assess the *in vivo* effects of the extract of the medicinal mushroom, *Lactarius deterrimus*, when administered (60 mg/kg, i.p.) daily for four weeks to streptozotocin- (STZ-) induced diabetic rats. Diabetic rats treated with the *L. deterrimus* extract displayed several improved biochemical parameters in the circulation: reduced hyperglycemia, lower triglyceride concentration and reduced glycated hemoglobin, glycated serum protein, and advanced glycation end product (AGE) levels. This treatment also adjusted the diabetes-induced redox imbalance. Thus, higher activities of the antioxidative enzymes, superoxide dismutase, and catalase in the circulation were accompanied by increased levels of free intracellular thiols and glutathionylated proteins after treatment with the *L. deterrimus* extract. In addition to a systemic antioxidant effect, the administration of the extract to diabetic rats also had a positive localized effect on pancreatic islets where it decreased AGE formation, and increased the expression of chemokine CXCL12 protein that mediates the restoration of *β*-cell population through the activation of the serine/threonine-specific Akt protein kinase prosurvival pathway. As a result, the numbers of proliferating cell nuclear antigen- (PCNA-) and insulin-positive *β*-cells were increased. These results show that the ability of the *L. deterrimus* extract to alleviate oxidative stress and increase *β*-cell mass represents a therapeutic potential for diabetes management.

## 1. Introduction

Diabetes mellitus (DM) is a metabolic disorder caused by absolute insulin deficiency or insufficient insulin secretion and/or insulin sensitivity [1] and is characterized by hyperglycemia. Pancreatic *β*-cells are responsible for insulin production and maintenance of blood glucose concentrations [2]. *β*-cell dysfunction and declining *β*-cell numbers are responsible for the loss of endocrine pancreas function in both type 1 (T1D) and type 2 diabetes, albeit loss of *β*-cells is more rapid in autoimmune destruction in T1D. Hyperglycemia is at the root of the significant increase in the formation of toxic reactive oxygen species (ROS) and establishment of oxidative stress that is responsible for the progression of diabetes and its complications [1], with vascular injury, hypertension, nephropathy, retinopathy, and neuropathy as major outcomes.

Despite many strategies and agents, DM management requires constant improvement. Considering that synthetic drugs have specific limitations in treating diabetic complications, it is important to refine approaches based on novel natural compounds that could support the restoration and maintenance of pancreatic *β*-cell numbers and assist in controlling diabetic complications [3–5]. Since oxidative stress is an essential contributor to the development and progression of diabetes and related complications, the therapeutic approach to DM is based on improving hyperglycemia and the organism's endogenous antioxidant activities. Recent investigations that have provided evidence for the antidiabetic effectiveness of mushrooms provide novel approaches for controlling DM and its complications [6]. Edible mushrooms are rich in vitamins (B, D, A, C, K), contain high protein contents and minerals, and are low in saturated fats. Many of the biological characteristics of mushrooms are mainly due to the presence of dietary fiber, in particular chitin and the *α* and *β* glucans that have significant nonspecific immunostimulatory effects [7, 8]. Mushrooms have also proven to be effective ROS scavengers, their antioxidant properties correlating with their total content of phenolics [4, 9, 10].

*Lactarius deterrimus*, also known as false saffron milkcap, is an edible mushroom from the family Russulaceae that mainly grows in coniferous woods in northern, northeastern, and Central Europe.* L. deterrimus *and other* Lactarius* species, such as* Lactarius salmonicolor*,* Lactarius deliciosus*, and* Lactarius sanguifluus*, possess potent medicinal activities. The antimicrobial activities against several Gram (+) and Gram (−) bacteria and the anticancer and antiviral activities of their antioxidant constituents have been described; in addition, it has been suggested that these mushrooms represent a potential source of natural immunostimulatory substances [11–13]. Previously, we described the* in vitro* antioxidant and scavenging properties of the* L. deterrimus* extract, especially NO-scavenging and metal-chelating activities, which correlated with the ability of the extract to prevent lipid peroxidation and DNA damage during streptozotocin-induced oxidative stress in Rin-5F cells [14].

The aim of this study was to examine the potential* in vivo* beneficial effect of the extract obtained from the edible mushroom* Lactarius deterrimus* on the systemic antioxidant status and control of pancreatic damage in streptozotocin- (STZ-) induced diabetic rats.

## 2. Materials and Methods

### 2.1. Mushroom Collection and Extract Preparation

The mushroom* Lactarius deterrimus* was collected near the village Mune from the Istra region in Croatia in the summer of 2008. Fruiting bodies were gently cleansed of any residual compost. Fresh mushrooms were air-dried and stored in airtight plastic bags at room temperature. The dried mushroom samples were broken up in a blender before extraction with 50% ethanol at a sample : solvent ratio of 1 : 10 (w/v). The extraction process was carried out using an ultrasonic bath (B-220; Branson and SmithKline Company) at 45°C for 40 min. After filtration, the extraction solvent was removed by a rotary evaporator (Devarot; Elektromedicina) under vacuum. The obtained* L. deterrimus* extracts (Ld) were dried at 60°C to a constant mass and stored in glass bottles at −80°C to prevent oxidative damage.

### 2.2. Phytochemical Analysis of the* L. deterrimus* Extract

The total phenolic compounds and other oxidation substrates contained in dry mushroom extracts were determined by the Folin-Ciocalteu colorimetric method based on absorbance at 765 nm [15] and are shown in Table 1. The values are expressed as g of gallic acid equivalents (GAE) per 100 g of the dry mushroom extract sample. The total flavonoid content was analyzed by the aluminum-chloride colorimetric assay at 510 nm [16] and is expressed as g of quercetin equivalents (QE) per 100 g of the dry extract sample. Previous detailed qualitative and quantitative analyses of the extract by high-performance liquid chromatography with diode-array detection (HPLC/DAD) revealed the presence of tryptophan, p-hydroxybenzoic acid, and unsaturated oxy(hydroxyl- or epoxy-) fatty acids [14].

### 2.3. Animals

Experiments were performed on 2.5-month-old adult albino Wistar rats weighing 220–250 g. All animal procedures were in compliance with the EEC Directive (86/609/EEC) on the protection of animals used for experimental and other scientific purposes and were approved by the Ethical Committee for the Use of Laboratory Animals of the Institute for Biological Research “Siniša Stanković”, University of Belgrade.

### 2.4. Experimental Design

The experimental model of multiple low dose (MLDS) STZ-induced diabetes was used. Diabetes was induced by injection (i.p.) of STZ (40 mg/kg/day) to Wistar rats for five consecutive days. STZ was dissolved before use in sodium citrate buffer (0.1 M, pH 4.5). Blood glucose was measured 24 h after the last STZ injection. Blood samples were obtained from the tail vein of rats that fasted overnight, and glucose was measured with a blood glucose meter (Accu-Chech Active). Rats were considered to have diabetes when the fasting blood glucose level exceeded 20 mmol/L (the baseline glucose level measured before diabetes induction by STZ was 6.2 ± 0.3). Male albino Wistar rats were randomly divided into four groups: (i) NDM: the nondiabetic group (*n* = 7), also referred to as the negative control, received citrate buffer (i.p.) equivalent to the STZ injection for 5 consecutive days; (ii) NDM + Ld: the nondiabetic group (*n* = 7), also referred to as the positive control, was administered the mushroom extract (Ld; 60 mg/kg, i.p.) daily for four weeks; (iii) DM: the diabetic group (*n* = 8) was injected STZ and left untreated throughout the four week period; (iv) DM + Ld: the diabetic group (*n* = 8) was treated with the Ld daily for four weeks, starting from the last day of STZ administration.

### 2.5. Serum and Hemolysate Preparation

Rat blood serum was collected after blood clotting and centrifugation at 2000 ×g for 10 min. The serum was used for determination of glycated proteins and the amounts of protein sulfhydryl groups (–SH). For the preparation of red blood cell (RBC) hemolysates, blood was collected in heparinized tubes (1 000 IU heparin) and centrifuged at 2000 ×g for 10 min. RBCs were washed twice with 0.9% NaCl and centrifuged. The washed RBCs were lysed in 3 volumes of cold water for 30 min on ice. Hemolysates were used for the determination of catalase (CAT), superoxide dismutase (SOD), glutathionylated proteins (GSSP), hemoglobin (Hb), and glycated (Gly) Hb.

### 2.6. Determination of Biochemical Parameters of Diabetes

Blood glucose levels were measured with a commercial kit (Gluco-quant Glucose/HK, Boehringer Mannheim, Germany) based on the hexokinase/G6P-DH enzymatic method. GlyHb was determined by the colorimetric assay according to Parker et al. [17]. Serum triglycerides were measured by the glycerol-3-phosphate (GPO) oxidase-p-aminophenazone (PAP) method with an enzymatic kit (Randox Laboratories, UK). Detection of glycated serum proteins was by the fructosamine assay. The glycation of serum proteins was measured according to Johnson et al. [18]. Aliquots of sera (50 *μ*L) were added to 450 *μ*L of 100 mM carbonate buffer (pH 10.8) containing 0.5 mM nitro blue tetrazolium (NBT). The samples were incubated for 1 h at 37°C. Absorbance was measured at 595 nm.

### 2.7. Determination of GSSP in the Circulation

Acid-precipitated proteins in hemolysates were thoroughly washed with the precipitating solution until no trace of soluble reduced glutathione (GSH) or glutathione disulfide (GSSG) was detected. The pellets were then resuspended and brought to an alkaline pH (pH 7.5–8) for 5–30 min. Under these conditions, GSH is released via an –SH/disulfide (–S–S–) exchange reaction. The reaction was stopped by the addition of trichloroacetic acid to a final 5% concentration. The amount of released GSH was determined enzymatically in the supernatants after centrifugation.

### 2.8. Determination of Protein Sulfhydryl Groups in Circulation

Protein –SH groups were determined by Ellman's method [19]. Briefly, 0.5 mL of serum was added to a cuvette containing 0.1 M phosphate buffer, pH 7.4 (0.5 mL); 0.2 mL of 3 mM 5,5′-dithiobis (2-nitrobenzoic acid) was then added to start the reaction. Absorbance was measured at 412 nm after 10 min. The number of –SH groups was calculated according to the following formula:

(1) mol –SH/g  proteins=[A  sample14150×dilution  factor]/g proteins.

### 2.9. Determination of SOD and CAT Activities in the Circulation

CAT activity was determined according to Beutler [20] as the rate of hydrogen peroxide decomposition and expressed as *μ*mol H_2_O_2_/min/g Hb. The Hb was removed from hemolysates prior to measurement of SOD activity by the epinephrine method [21]. One unit of SOD activity was defined as the amount of Hb that causes 50% inhibition of adrenaline autoxidation.

### 2.10. Determination of AGE in Circulation

The fluorescent products of AGE were detected according to Munch et al. [22], Henle et al. [23], and Kalousová et al. [24]. Blood serum was diluted 1 : 50 with phosphate buffered saline (PBS) pH 7.4 and fluorescence intensity was measured at an excitation wavelength of 350 nm and an emission wavelength of 440 nm with a luminescence spectrometer (LS50B; Perkin-Elmer Ltd., Buckinghamshire, England). The fluorescence intensity was expressed in arbitrary units.

### 2.11. Histological Analysis and Immunostaining

The pancreata from all experimental groups were removed and fixed in 10% buffered formalin for histological and immunohistological examination. Pancreatic tissues from all examined groups were blocked in paraffin and sectioned at 5 *μ*m thickness for histological and immunohistochemical examination (Leica DMLB; objective magnification 40x). For histological analysis tissue sections were stained with hematoxylin and eosin and observed under a light microscope. For immunohistochemical analysis, deparaffinized sections were passed through xylene and rehydrated in sequentially graduated ethyl alcohol. Slides were incubated in 0.3% hydrogen peroxide/methanol for 20 min to reduce nonspecific background staining due to endogenous peroxidase. After washing in PBS, the sections were treated with 0.01 M sodium citrate buffer at 98°C for antigen retrieval. The cooled tissues were washed four times in PBS prior to application of the blocking serum for 5 min (0.05% Tween 20, 5% bovine serum albumin). The primary antibody was applied overnight at room temperature (RT). Polyclonal antibodies raised against insulin, CXCL12, C-X-C chemokine receptor type 4 (CXCR4), N(*ε*)-(carboxymethyl)lysine (CML), an advanced glycation end product (AGE), receptor for AGE (RAGE), phosphorylated protein kinase B (pAkt), and proliferating cell nuclear antigen (PCNA) (Santa Cruz Biotechnology, Santa Cruz, CA, USA) were diluted 1 : 50 in PBS with 2% dry skimmed milk. After washing in PBS, sections were incubated at RT for 1 h with secondary antibody horseradish peroxidase (1 : 100) (Santa Cruz Biotechnology, Santa Cruz, CA, USA). Tissues were incubated for 20 min at RT in a solution of 3,3′-diaminobenzidine (DAB). After washing with PBS, the tissues were counterstained with haematoxylin and washed in water, and the coverslips were applied with mounting media. For the negative control, the primary antibody was not added to the sections.

### 2.12. Statistical Analysis

The data were expressed as the mean ± S.E.M. (standard error of mean). Statistical differences between groups were analyzed using one-way analysis of variance (ANOVA), followed by Duncan's multiple range test. The difference was considered statistically significant at *P* < 0.05.

## 3. Results

### 3.1. Biochemical Parameters of Diabetes in Rats Treated with the* L. deterrimus* Extract

**Figure 1:**
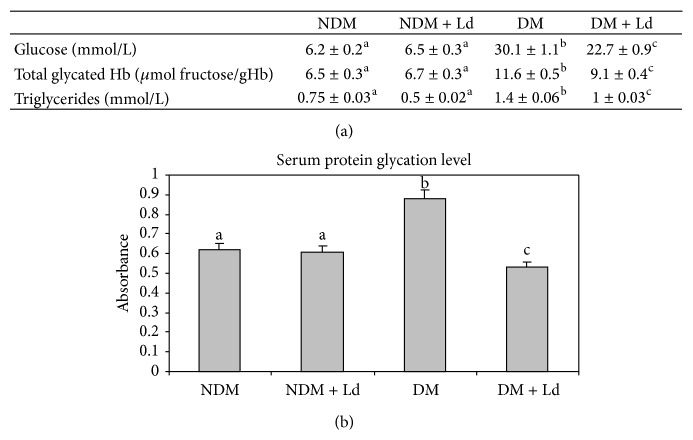
The effect of* L. deterrimus* extract administration on the biochemical parameters in sera (a) and glycation levels of serum proteins (b). NDM: control rats; NDM + Ld: control rats treated daily with* L. deterrimus* extract for four weeks; DM: STZ-induced diabetic rats; DM + Ld: STZ-induced diabetic rats treated with* L. deterrimus* extract for four weeks. Hb: haemoglobin. The values are presented as the mean ± S.E.M.; values not sharing a common superscript letter differ significantly at *P* < 0.05.

Administration of Ld led to an overall improvement of the main biochemical parameters of diabetes, albeit short of their restoration to their respective physiological levels. As can be seen in Figure 1(a), treatment of diabetic animals with Ld reduced the glucose concentration by almost 25% and lowered the level of triglycerides by about 28% and GlyHb by about 21%. The treatment also lowered the level of serum protein nonenzymatic glycation to the level measured in control rats (Figure 1(b)). Ld-treated nondiabetic rats did not exhibit any changes of these parameters when compared to the control group.

### 3.2. Oxidative Stress and Antioxidative Protection in the Circulation of Rats Treated with the* L. deterrimus* Extract

**Figure 2:**
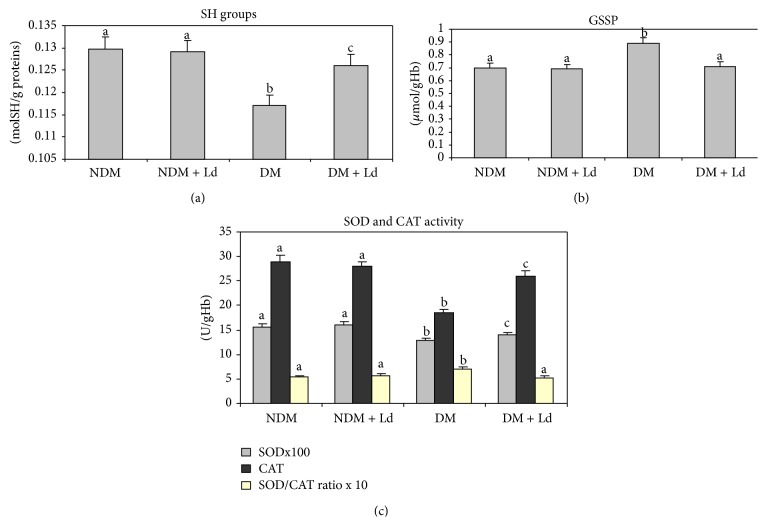
The effect of* L. deterrimus* extract administration on the content of free –SH (a), level of protein bound glutathione (GSSP) (b), and activities of antioxidative enzymes (c) in the circulation. NDM: control rats; NDM + Ld: control rats treated daily with* L. deterrimus* extract for four weeks; DM: STZ-induced diabetic rats; DM + Ld: STZ-induced diabetic rats treated with* L. deterrimus* extract for four weeks. The values are presented as the mean ± S.E.M.; values not sharing a common superscript letter differ significantly at *P* < 0.05.

The redox status in experimental rats was estimated by measuring the levels of free intracellular thiols (Figure 2(a)) and GSSP (Figure 2(b)) that are directly linked to the redox state of the cell [25, 26]. These experiments revealed that the treatment with Ld significantly improved the redox parameters in the circulation. While the level of serum protein –SH groups was significantly decreased in diabetic rats, the treatment with Ld restored the –SH content almost to the control level. The –SH content was not significantly changed when control rats were administered the extract. As can be observed in Figure 2(b), the 1.3-fold increase in the level of GSSP in diabetic rats as compared to the control level was brought to the control level by the administration of Ld. The GSSP level was not significantly changed when healthy rats were administered Ld.

The activities of the major antioxidant enzymes, SOD and CAT, provide the first line of antioxidant defenses and protect cells from ROS damage [27]. As can be seen in Figure 2(c), the decreased levels of SOD and CAT activities in the circulation of diabetic rats were improved after Ld administration, the effect of Ld being more pronounced on CAT activity. In addition, a significant increase in the SOD/CAT ratio was observed under diabetic conditions, while the Ld treatment reduced this ratio to the control level in diabetic animals. SOD and CAT activities did not change after Ld administration to control rats.

### 3.3. *L. deterrimus* Extract Administration Induces Histological Changes and Stimulates the Production of Insulin and CXCL12 in Pancreatic Islets

**Figure 3:**
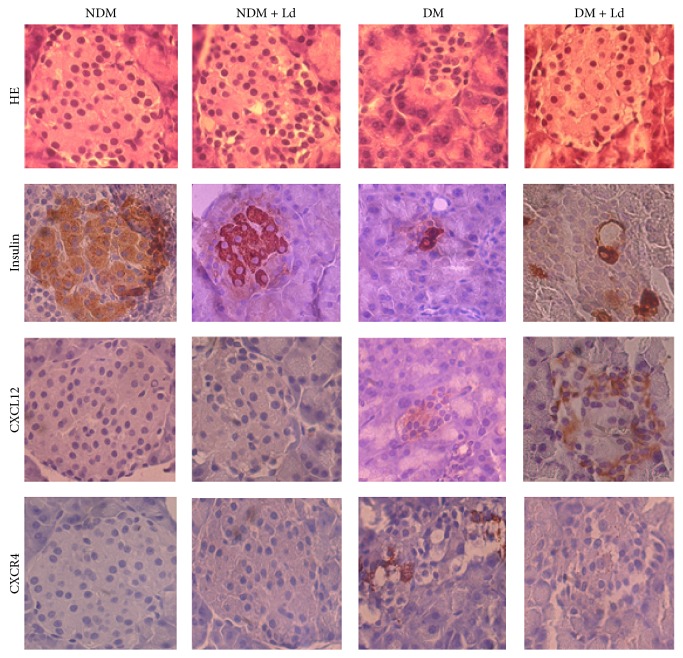
The effect of administration of the* L. deterrimus* extract on histological changes and immunohistochemical localization of insulin and CXCL12 in pancreatic islets. HE: hematoxylin and eosin staining of pancreatic sections; light photomicrographs of insulin, CXCL12, and CXCR4 immunohistochemical staining of pancreatic sections within islets (magnification 40x). NDM: control rats; NDM + Ld: control rats treated daily with* L. deterrimus* extract for four weeks; DM: STZ-induced diabetic rats; DM + Ld: STZ-induced diabetic rats treated with* L. deterrimus* extract for four weeks.

Hematoxylin and eosin sections (Figure 3) show that the pancreas of control rats is comprised of numerous, compactly arranged cells in the islets of Langerhans that appear as dense cords. The pancreas of diabetic rats had smaller pancreatic islets with lower numbers of *β*-cells, displaying increased vacuolation and clumped cells. Ld-treated diabetic rats resembled more closely normal islet cell architecture, which is suggestive of a protective role of Ld on the pancreas of diabetic rats (Figure 3, HE). Immunohistochemical staining with insulin revealed disorganized islets in diabetic rats, with unevenly distributed insulin-positive cells in comparison to control islets that displayed strong insulin immunostaining (Figure 3, insulin). The Ld treatment increased the number of insulin-positive cells in the islets of diabetic rats (Figure 3, insulin).

The chemokine CXCL12 and its receptor, CXCR4, have been shown to mediate *β*-cell repair [28]. Immunohistochemical staining revealed the presence of CXCL12-positive cells only in diabetic islets and their increased presence in islets from Ld-treated rats. As can be seen in Figure 3, CXCR4-positive immunostaining was only detected in the islets of diabetic rats.

### 3.4. The Effects of the* L. deterrimus* Extract Administration on CML-Modified Proteins in Pancreatic Islets

**Figure 4:**
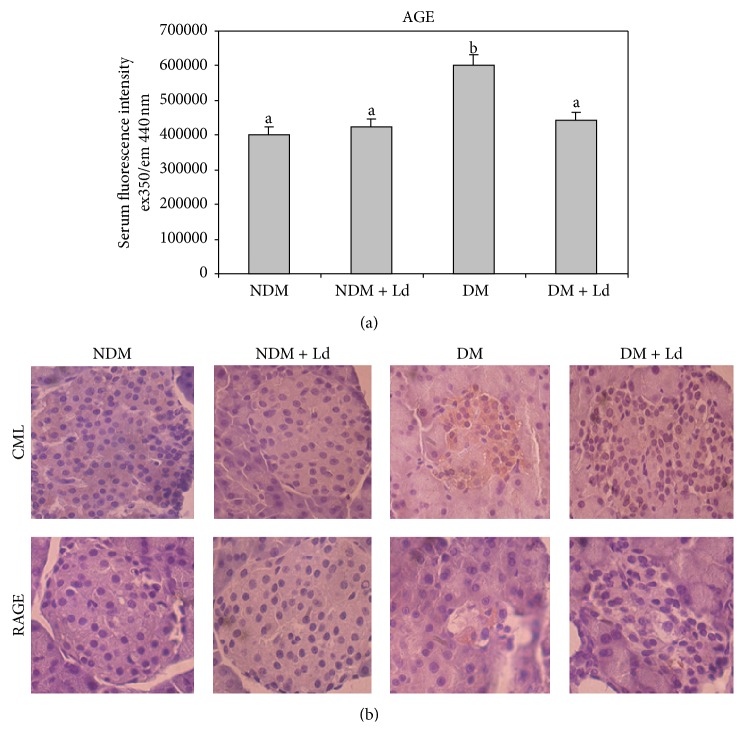
The effect of* L. deterrimus *administration on AGE in the circulation (a) and presence of CML-modified proteins in pancreatic islets (b). Fluorescent products of AGE in the serum (a). Light photomicrographs of immunohistochemical staining for CML and RAGE of pancreatic sections within the islet (b) (magnification 40x). NDM: control rats; NDM + Ld: control rats treated daily with* L. deterrimus* extract for four weeks; DM: STZ-induced diabetic rats, DM + Ld: STZ-induced diabetic rats treated with* L. deterrimus* extract for four weeks. The values are presented as the mean ± S.E.M.; values not sharing a common superscript letter differ significantly at *P* < 0.05.

Chronic hyperglycemia causes tissue damage that is mediated in part by the nonenzymatic glycation and oxidation of proteins and lipids and the formation of AGE of which CML is one of the most often used markers [29]. AGE exert their effects through interactions with their receptor RAGE that is normally expressed at low levels on the surface of most cell types. In addition to the circulation, AGE accumulate in tissues where they contribute to the development of diabetic complications. In the pancreas they play a part in progressive *β*-cell loss [30]. Determination of the fluorescent products of AGE in the serum revealed a 1.5-fold increase in AGE in diabetic rats. In diabetic rats administered Ld, the increase in AGE was at the control level (Figure 4(a)). Immunohistochemical staining revealed an extensive distribution of CML-positive cells in the islets of diabetic rats. In Ld-treated diabetic rats, the CML-positive cells were more disperse (Figure 4(b), CML). RAGE was observed only in the islets of diabetic rats (Figure 4(b), RAGE).

### 3.5. The Effects of* L. deterrimus* Extract Administration on the Prosurvival and Proliferative Pathways in Pancreatic Islets

**Figure 5:**
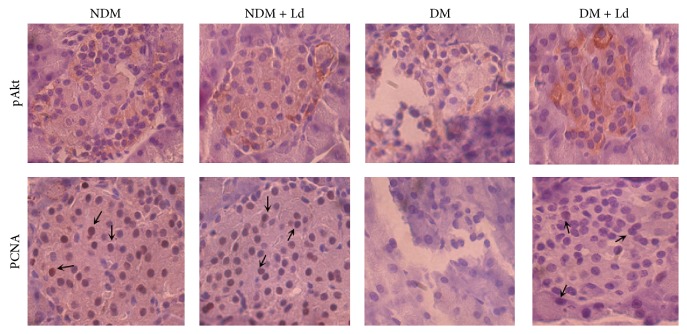
The effect of* L. deterrimus* extract administration on the prosurvival pathway and *β*-cell proliferation in pancreatic islets. Light photomicrographs of immunohistochemical staining for pAkt and PCNA of pancreatic sections within the islet (magnification 40x). NDM + Ld: control rats treated daily with* L. deterrimus* extract for four weeks; DM: STZ-induced diabetic rats; DM + Ld: STZ-induced diabetic rats treated with* L. deterrimus* extract for four weeks.

Phosphoinositide 3-kinases (PI3Ks) and its downstream effector protein kinase Akt mediate cellular survival signals that have an essential function in pancreatic *β*-cell survival [31]. Immunohistochemical staining with activated pAkt shown on Figure 5 revealed its presence in the islets of both control groups, whereas in diabetic islets pAkt-positive staining was weaker. Treatment of diabetic animals with Ld caused a considerable increase in pAkt staining that points to the stimulation of the prosurvival pathway in islets.

Immunohistochemical staining with PCNA (Figure 5), which assumes an essential function in DNA replication and repair, revealed its extensive distribution in the nuclei of islets of both control groups. However, in the islets of diabetic animals, no PCNA immunostaining was detected. The Ld-treated diabetic rats exhibited PCNA staining in their islets (Figure 5, PCNA). The increased presence of PCNA in Ld-treated diabetic rats points to the activation of proliferative mechanisms, which is in agreement with the described stimulation of the prosurvival pathway.

## 4. Discussion

In this report we have presented evidence for the beneficial effect of the daily i.p. administration for four weeks of the extract of* Lactarius deterrimus* (Ld) to STZ-induced diabetic rats. This was manifested as improved hyperglycemia and a resulting decline in GlyHb, serum protein glycation, and decreased formation of AGE in the circulation, as well as lower triglyceride concentrations. We interpret these global changes as part of a systemic antioxidant effect that impacted pancreatic islets in diabetic rats. By suppressing the formation and accumulation of potential inducers of *β*-cell damage in diabetic rats (detected as a decrease in CML-containing species in the pancreas), Ld administration activated prosurvival CXCL12/Akt signaling and the proliferative pathway, observed as the increased presence of PCNA-containing *β*-cells. Therefore, by shifting the balance of *β*-cell death in favor of *β*-cell survival, the net result of Ld administration was an increase in the number of functional insulin-positive *β*-cells.

High concentrations of ROS are involved in pathological changes of cellular functions and disruptions of cellular homoeostasis [1]. In diabetes, persistent hyperglycemia causes enhanced glucose autooxidation and protein glycosylation that increase the production of ROS that are important agents in the development of diabetic complications. The lower activities of the antioxidant enzymes CAT and SOD, along with the decreased protein –SH content and increased amounts of GSSP moieties, serum protein glycation, and GlyHb, revealed the presence of oxidative stress in the circulation of diabetic rats. After the four-week treatment of diabetic rats with Ld, these parameters were practically restored to their physiological values. Ld administration caused a significant decrease in the SOD/CAT ratio that was associated with lower hydrogen peroxide levels, decreased oxidative stress. This shows that the administration of Ld under diabetic conditions attenuated the oxidative stress-induced harmful processes by improving hyperglycemia and promoting increased activities of antioxidative enzymes.

These results are consonant with literature data showing that edible mushrooms possess antioxidant and free radical scavenging properties [3]. The Ld applied herein is enriched in both phenolics and flavonoids that contributed to its free-radical scavenging activity (Table 1) [14]. This assumption is in agreement with the generally held view that the antioxidant properties of mushroom extracts correlate with their total content of phenolics [10] and with Wang and Xu [5] who recently provided additional evidence that phenols and polyphenols are the major naturally occurring antioxidant compounds in wild edible mushrooms. We speculate that the free radical scavenging activity of Ld could also be attributed to *β*-glucan, a principal component of mushrooms known to play important roles in the activation of the nonspecific immune response, reduction of blood cholesterol and blood glucose concentrations, protecting blood macrophages from ionizing radiation, and restoring bone marrow production [5, 32, 33]. Results from our previous work with plant-sourced *β* glucan revealed its ability to cause a systemic adjustment of redox disturbance and to exert a beneficial effect under diabetic conditions via its antioxidant and anti-inflammatory activities [34, 35].

AGE also play a role in toxic signaling in diabetic pathology by contributing to unbalanced free-radical formation and activating stress signaling in cells through interactions with RAGE. AGE are caused by glycation that involves a series of nonenzymatic reactions between the carbonyl group on reducing sugars and the amino group on proteins [36]. The classical pathway of AGE formation involves a glucose-protein condensation reaction to form Schiff base adducts that undergo Amadori rearrangement [37]. The early glycosylation products accumulate predominantly on long-lived proteins that undergo a series of* in vivo *rearrangements to form irreversible compounds and a number of reactive intermediates that enhance oxidative stress [38]. Our results describe a significant increase in serum AGE in diabetic rats and their reversion to the control level after the Ld treatment. Through the generation of ROS and reactive nitrogen species, AGE contribute to tissue injury by changing the structure and function of proteins, thereby affecting important cellular functions, either directly or via RAGE-activated pathways [39]. In addition to AGE in the circulation, we established the presence of CML, the most abundant AGE* in vivo*, and of RAGE in pancreatic islets of diabetic rats. Importantly, in pancreatic islets of Ld-treated diabetic animals, CML was barely detectable and RAGE was undetectable by the experimental procedure applied herein. AGE generally accumulate in tissues that display diabetic complications [24, 40]. Lee and coworkers [30] have demonstrated that the interaction between AGE and RAGE contributes to the progressive loss of *β*-cells in diabetes through intracellular ROS generation. This finding and our results indicate that the *β*-cells of Ld-treated diabetic rats were exposed to a lower level of toxic signals than *β*-cells of diabetic rats.

The efficacy of medicinal mushrooms in the treatment of diabetes by protecting against *β*-cell damage through enhanced antioxidant defenses, reduced inflammation, and increased insulin release has been reported [6, 41, 42]. The results obtained herein by immunohistological examination of the pancreas revealed that Ld administration to diabetic rats restrained islet destruction and partially restored the number of insulin-positive cells. The activation and direct involvement of the chemokine CXCL12/CXCR4 receptor pair in cardiomyocytes and pancreatic islets have been demonstrated after tissue injury [43, 44], the latter authors showing increased CXCR4 mRNA and protein expression in insulitis. Recently it was established that injury of *β*-cells induces CXCL12 expression that initiates the transdifferentiation of *α*-cells to *β*-cells in the pancreas [45]. PI 3-kinase and the downstream effector protein Akt play key roles in mediating signals for cell growth, cell survival, cell-cycle progression, and differentiation [31, 46]. Activated, phosphorylated Akt by insulin and growth factors is implicated in glucose metabolism, transcriptional control, and mediation of antiapoptotic and prosurvival events in *β*-cells [47, 48]. Yano et al. [49] described the important function of CXCL12 in diabetes attenuation in mice via promotion of *β*-cell survival by Akt activation. The functions of CXCL12 described in the literature suggested to us that the increased presence of CXCL12 stimulated the Akt pathway, affecting elevated expression of PCNA protein in Ld-treated diabetic rats. This result indicates that Ld exerted a stimulatory effect on islet proliferation and regeneration. Considering the significant capacity of the endocrine pancreas to adjust to changes in insulin demand [50], and that the pancreas contains quiescent cells that can proliferate and replace lost cells [51], we believe that the described stimulatory activity of Ld can exert an important beneficial effect during the initial stage of diabetes development when it is potentially possible to expand the still existing *β*-cell mass through regeneration. Pancreatic *β*-cell regeneration induced by *β*-cell proliferation is often mentioned as a vital goal in the development of effective treatments for diabetes [52]. The findings described herein point to the possibility of developing the therapeutic potential of* Lactarius deterrimus* in diabetes management by employing its ability to alleviate oxidative stress and increase *β*-cell mass.

## 5. Conclusion

*In vivo* antioxidant and antidiabetic effects of the extract from the mushroom* Lactarius deterrimus* were determined. Our results show that administration of the extract to diabetic rats restores antioxidant enzyme activities in the circulation and decreases oxidative stress. Treatment with the extract exhibits a potential for preserving pancreatic islet structure and increasing *β*-cell mass through activation of the prosurvival CXCL12/Akt pathway and stimulation of *β*-cell proliferation. We believe that the effects of the mushroom extract are important as it is derived from an established food used for human consumption. However, as a caveat to these findings, the described potentially positive effects have to be proven in human diabetics, and any side effects from prolonged extract administration as described herein need to be assessed.

## Figures and Tables

**Table 1:** Phytochemical constituents of the *L. deterrimus* extract.

	Phenolic content (mg GAE/g extract)^a^	Flavonoid content (mg QE/g extract)^b^
*Lactarius deterrimus *	14.8 ± 2.23	5.07 ± 1.97

Total phenolic and flavonoid contents were determined by the Folin Ciocalteu and aluminium-chloride colorimetric methods, respectively, and are expressed as milligrams of gallic acid (GA) per gram of dry mushroom material^a^ and as milligrams of quercetin (QE) per gram of dry mushroom material^b^. Data are presented as means ± SD.
