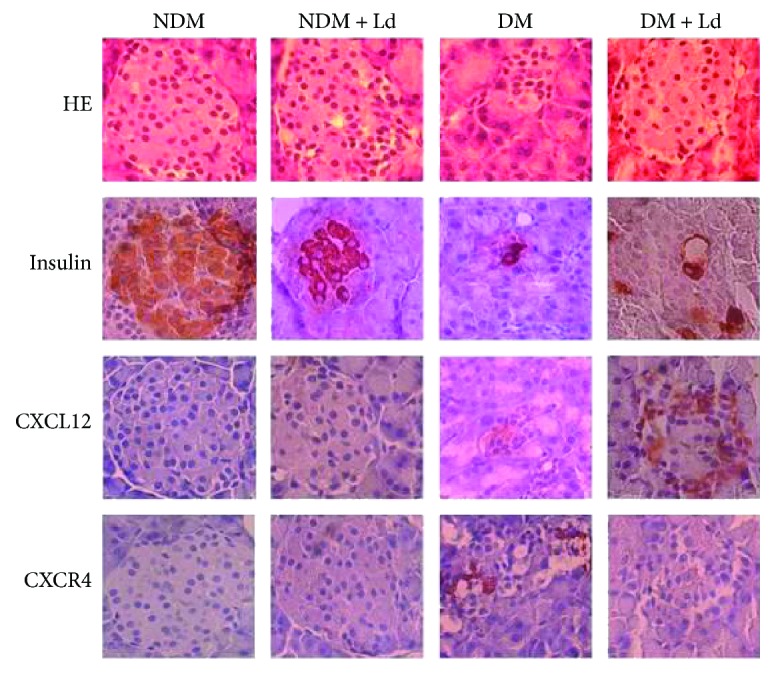# Corrigendum to “Protective Effects of the Mushroom *Lactarius deterrimus* Extract on Systemic Oxidative Stress and Pancreatic Islets in Streptozotocin-Induced Diabetic Rats”

**DOI:** 10.1155/2017/1638645

**Published:** 2017-12-20

**Authors:** Mirjana Mihailović, Jelena Arambašić Јovanović, Aleksandra Uskoković, Nevena Grdović, Svetlana Dinić, Senka Vidović, Goran Poznanović, Ibrahim Mujić, Melita Vidaković

**Affiliations:** ^1^Department of Molecular Biology, Institute for Biological Research, University of Belgrade, Bulevar Despota Stefana 142, 11060 Belgrade, Serbia; ^2^Department of Biotechnology and Pharmaceutical Engineering, Faculty of Technology, University of Novi Sad, Bulevar Cara Lazara 1, 21000 Novi Sad, Serbia; ^3^Biotechnical Faculty, University of Bihać, Kulina Bana 2, 77000 Bihać, Bosnia and Herzegovina

In the article titled “Protective Effects of the Mushroom *Lactarius deterrimus* Extract on Systemic Oxidative Stress and Pancreatic Islets in Streptozotocin-Induced Diabetic Rats” [1], there was an error in Figure 3, where the plate “NDM and CXCL12” should be replaced with the plate “NDM + Ld and RAGE” from Figure 4. Figure 3 should be corrected as follows.

## Figures and Tables

**Figure 1:**